# Postural control and trunk mobility impairments in adults with long COVID: A cross-sectional study using computerized posturography and clinical biomechanical tools

**DOI:** 10.1371/journal.pone.0354593

**Published:** 2026-08-13

**Authors:** Adel Alshahrani, Ravi Shankar Reddy, Kumar Gular, Venkata Nagaraj Kakaraparthi, Ajay Prashad Gautam

**Affiliations:** 1 Department of Medical Rehabilitation Sciences, Physical Therapy Program, College of Applied Medical Sciences, Najran University, Najran, Saudi Arabia; 2 Health Research Center, Najran University, Najran, Saudi Arabia; 3 Department of Medical Rehabilitation Sciences, Physical Therapy Program, College of Applied Medical Sciences, King Khalid University, Abha, Saudi Arabia; Universita Politecnica delle Marche Facolta di Ingegneria, ITALY

## Abstract

Long COVID is increasingly associated with persistent neuromuscular and functional impairments, including deficits in balance and trunk control. This cross-sectional study examined postural stability and trunk biomechanics in adults with Long COVID, comparing them to matched healthy controls. A total of 128 participants (64 per group) underwent computerized posturography to assess sway area, sway velocity, and stability index under varying sensory conditions. Trunk range of motion and isometric muscle strength were measured, along with fatigue, pain sensitivity, and physical function, using validated patient-reported outcomes. Compared to controls, individuals with Long COVID demonstrated significantly larger sway area (eyes closed on foam: 8.12 ± 1.87 cm^2^ vs. 6.01 ± 1.65 cm^2^, p < 0.001), higher sway velocity (1.23 ± 0.34 cm/s vs. 0.98 ± 0.29 cm/s, p < 0.001), and lower stability index (78.45 ± 5.67 vs. 83.21 ± 6.12, p < 0.001). Trunk flexion and extension were reduced (p < 0.001), as were flexor and extensor strength (p < 0.001). Fatigue was markedly elevated (FSS: 5.72 ± 1.03 vs. 2.34 ± 0.98, p < 0.001). Multivariate analysis identified trunk extensor strength (β = 0.34, p = 0.001), fatigue severity (β = –0.38, p = 0.002), and physical function (β = 0.28, p = 0.015) as independent predictors of postural instability. These findings underscore the need for integrated rehabilitation that addresses both trunk biomechanics and symptom burden in Long COVID.

## Introduction

The coronavirus disease 2019 (COVID-19) pandemic has caused significant and lasting health issues in a notable number of individuals, even after they recover from the initial infection [1]. Known as “Long COVID” or post-acute sequelae of SARS-CoV-2 infection (PASC), this condition involves a range of persistent symptoms—including fatigue, musculoskeletal pain, dizziness, and functional impairments—that can last for months or longer [2]. An emerging concern is how Long COVID affects neuromuscular and postural functions, especially given the increased risk of falls, disability, and reduced quality of life linked to balance problems [3]. Postural control, a complex sensorimotor process that integrates vestibular, visual, proprioceptive, and muscular inputs, can be disrupted in chronic health conditions and may be adversely affected in individuals recovering from COVID-19 [4]. Several biological mechanisms have been proposed to explain sensorimotor deficits in Long COVID, including persistent neuroinflammation, autonomic nervous system dysfunction, microvascular alterations, and impaired central sensory integration [5]. These pathophysiological changes may disrupt vestibular, proprioceptive, and neuromuscular processing, thereby contributing to deficits in postural regulation and balance performance [5].

Recent research has highlighted balance issues and gait changes in patients with Long COVID, with early evidence indicating deficits in proprioception and neuromuscular control [6]. Studies have demonstrated increased postural sway and decreased functional mobility in post-COVID individuals compared to matched controls [7,8]. Additionally, symptoms like fatigue and pain, which are commonly reported in this group, are known to contribute to reduced motor performance and balance [9]. While the physiological origins of these symptoms are under investigation, the role of biomechanical factors—particularly trunk mobility and strength—in postural instability in Long COVID has not been thoroughly explored [10]. Emerging evidence also suggests that persistent neuroinflammatory responses and central sensitization may contribute to prolonged fatigue, pain, and altered motor control in Long COVID, potentially affecting postural stability by altering sensorimotor processing and movement coordination [11]. Importantly, the trunk is vital for maintaining an upright posture and initiating balance adjustments, and dysfunction in this area may be a modifiable factor in balance decline [9].

Despite its clinical importance, the literature lacks comprehensive analyses that simultaneously examine objective postural control metrics, trunk biomechanical features, and subjective symptom burden in Long COVID [12]. Previous studies often evaluate these areas separately, which limits the ability to identify meaningful relationships or develop holistic rehabilitation strategies [12]. Specifically, the role of trunk strength and range of motion in balance impairments—when considered alongside self-reported fatigue, pain sensitivity, and functional limitations—has not been adequately quantified using objective and highly sensitive methods such as computerized posturography, which enables quantitative assessment of center-of-pressure displacement and subtle balance impairments, together with standardized symptom assessments.

Therefore, the current study aimed to (1) assess and compare static postural control in adults with Long COVID and age- and sex-matched healthy controls using computerized posturography; (2) evaluate trunk mobility and isometric trunk muscle strength in individuals with Long COVID and explore their relationship with postural control; and (3) examine correlations between postural control parameters and patient-reported fatigue, pain sensitivity, and physical function. It was hypothesized that individuals with Long COVID would demonstrate significantly impaired postural control, reduced trunk mobility and strength, and that these impairments would be closely associated with greater symptom burden and poorer balance performance.

## Methods

### Study design

This prospective cross-sectional study was conducted from 12/07/2024–30/05/2025 at the Balance and Movement Analysis Clinic in Abha, Aseer, Saudi Arabia. Ethical approval was granted by the institutional review board (REC# 234–2025) of DRS, and written informed consent was obtained from all participants before enrollment, ensuring full adherence to the principles outlined in the Declaration of Helsinki.

### Participants

**Fig 1:**
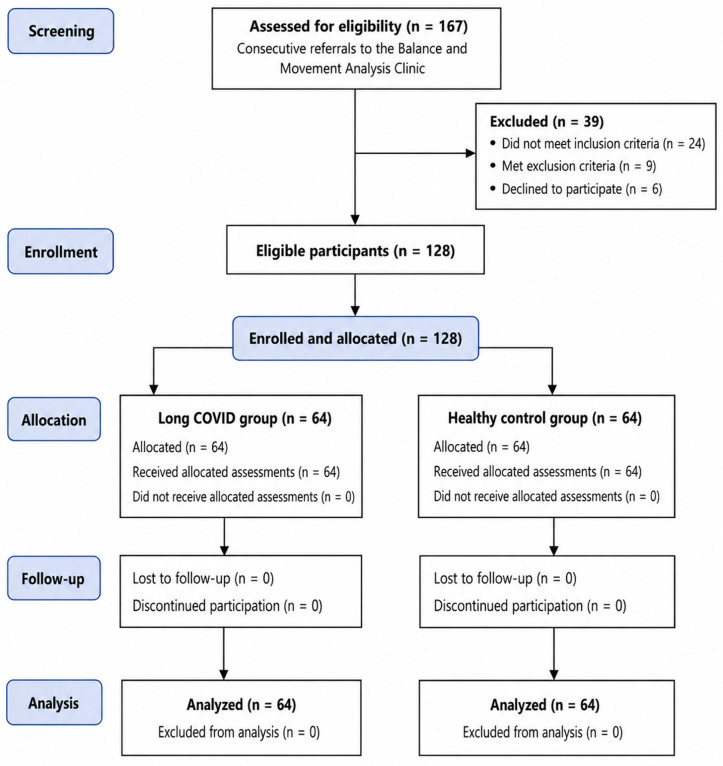
A participant flow diagram illustrating screening, exclusion, enrollment, and analysis.

Participants were recruited consecutively from outpatient referrals to the Balance and Movement Analysis Clinic, aged 18–65 years, with a confirmed history of SARS-CoV-2 infection at least 12 weeks prior to enrollment. During the recruitment period, 167 individuals were screened for eligibility. Of these, 24 did not meet the inclusion criteria, 9 met one or more exclusion criteria, and 6 declined participation. The remaining 128 participants were enrolled and completed all assessments, comprising 64 individuals with Long COVID and 64 healthy controls. A participant flow diagram illustrating screening, exclusion, enrollment, and analysis is presented in Fig 1. No participants withdrew after enrollment or had incomplete outcome data. They also had to have ongoing symptoms consistent with the World Health Organization’s clinical definition of Long COVID. Diagnosis was verified using medical records and structured clinical interviews. Participants were screened to confirm the presence of persistent fatigue, post-exertional symptoms, or musculoskeletal complaints consistent with post-viral sequelae. Inclusion criteria required participants to be independently ambulatory and medically stable, with no acute illness or hospitalization within the previous month. Exclusion criteria included any history of pre-existing neurological disorders; vestibular or balance impairments unrelated to COVID-19; musculoskeletal conditions that significantly affect posture or gait; cognitive impairments that limit test participation; or current involvement in physical rehabilitation programs. Control participants were age- and sex-matched healthy adults without a history of COVID-19, recruited from the university community through advertisements and screened to ensure they did not experience chronic fatigue, pain, or balance-related issues. All participants underwent eligibility confirmation through structured clinical screening and baseline assessments of demographics and health before enrollment.

### Postural control assessment

The primary outcome variable in this study was postural control, measured quantitatively using computerized static posturography with the TecnoBody IsoFree stabilometric force platform [1] (TecnoBody S.r.l., Italy). This high-precision system features multiple load cells and proprietary software that track the real-time displacement of the center of pressure (COP), providing validated, sensitive indices of postural control [13]. Three key posturography parameters were recorded: sway area (cm^2^), sway velocity (cm/s), and the stability index, each representing different aspects of postural control [14]. Sway area measures the two-dimensional range of COP movement; sway velocity indicates the rate of COP movement over time; and the stability index is a composite score generated by IsoFree software, with lower values indicating poorer postural control. The stability index is a dimensionless composite score generated by the IsoFree system and reflects overall balance performance based on center-of-pressure displacement characteristics. All participants were tested under four standardized sensory conditions: eyes open and eyes closed on both firm and foam surfaces, based on the modified Clinical Test of Sensory Interaction on Balance (mCTSIB). These conditions selectively challenge visual, proprioceptive, and vestibular systems, helping identify sensory integration issues. During each trial, participants stood barefoot with their feet aligned on standardized markers, arms relaxed, and gaze fixed at eye level during eyes-open trials. Each trial lasted 30 seconds, with a 60-second rest between trials to reduce fatigue. A familiarization trial was conducted before data collection. The platform was calibrated before each session following the manufacturer’s instructions. To ensure consistency and reduce bias, all assessments were performed by a single examiner who was blinded to the group allocation, in a quiet, temperature-controlled laboratory. The order of sensory conditions was randomized to control for sequence effects. Raw data were exported for offline analysis, and only valid, artifact-free trials were included in the final results.

### Trunk mobility

Trunk mobility was assessed through active range of motion (ROM) testing in the sagittal and transverse planes, specifically including trunk flexion and extension [15]. Measurements were obtained using a dual digital inclinometer (Baseline Bubble Inclinometer, Fabrication Enterprises Inc., USA), a validated instrument for assessing spine mobility in clinical research [16]. The dual inclinometer has demonstrated excellent intra-rater reliability for lumbar spine range-of-motion assessment in previous studies, with reported intraclass correlation coefficients exceeding 0.85 [2]. Intra-rater reliability was not specifically evaluated in the present study; however, all measurements were performed by the same trained examiner using standardized procedures to maximize consistency. Participants were instructed to stand upright with their feet shoulder-width apart and their arms crossed over their chests to prevent compensation. For trunk flexion and extension, the inclinometer units were placed at T12–L1 and S1 to ensure accurate segmental motion capture while minimizing pelvic involvement. Three trials were performed for both trunk flexion and extension, with the mean value recorded in degrees. A trained examiner provided standardized verbal instructions and visual demonstrations to ensure that participants maintained correct positioning and consistent technique. Rest intervals were allowed between trials to avoid fatigue. The same examiner conducted all tests and was blinded to group allocation, reducing inter-rater variability and minimizing measurement bias.

### Trunk isometric muscle strength

Trunk isometric muscle strength was evaluated using the Hoggan Scientific MicroFET2 Handheld Manual Muscle Tester, a validated tool for quantifying isometric force in musculoskeletal assessment [17,18]. Participants were positioned on an adjustable plinth with the trunk secured at two distinct angles using padded stabilization straps to minimize compensatory movement. For trunk flexor testing, the backrest was inclined to approximately 120°, and for extensor testing, it was adjusted to a near-vertical 90° position. In both positions, additional stabilization belts were applied across the thighs and upper torso to ensure isolation of the trunk musculature. The handheld dynamometer was placed against the participant’s sternum (for flexor testing) or upper thoracic spine at the T6–T8 level (for extensor testing). It was firmly secured using an overlaid strap to maintain consistent positioning. Participants were instructed to exert maximal isometric force against the dynamometer, maintain their posture, and avoid accessory movements. Each contraction was held for 5 seconds and repeated 3 times. Consistent with standard dynamometry procedures, the highest force output (in Newtons) obtained across the three trials was used for analysis. This differed from the trunk range-of-motion assessment, for which the mean value of three trials was recorded. Standardized verbal instructions and rest intervals were provided, and a trained, blinded examiner conducted all assessments to ensure methodological consistency and intra-rater reliability.

### Independent variables

The primary independent variable in this study was group allocation, defined as either Long COVID or healthy control. Participants in the Long COVID group met the World Health Organization criteria for post-COVID-19 condition, which include symptoms that persist for more than 12 weeks after a confirmed SARS-CoV-2 infection and are not explained by an alternative diagnosis. Healthy controls were age- and sex-matched individuals with no history of COVID-19 or of neurological, musculoskeletal, or cardiopulmonary conditions. Group assignment was confirmed through structured clinical interviews and validated symptom questionnaires. In addition, demographic characteristics such as age, sex, height, weight, and body mass index (BMI) were included as independent variables to control for anthropometric influences on balance and strength parameters.

### Covariates and confounders

Several clinically relevant covariates were incorporated to account for potential confounding factors influencing postural control and neuromuscular function. Covariates were selected a priori based on evidence from previous studies demonstrating associations between fatigue, pain, physical function, and postural control in individuals with Long COVID and related chronic conditions, as well as their clinical relevance as potential confounders of neuromuscular performance and balance outcomes. Selection was not based on statistical significance in preliminary bivariate analyses. These included self-reported fatigue severity, pain pressure thresholds, pain interference scores, and overall physical function. Fatigue was measured using the Fatigue Severity Scale (FSS) [19] and the PROMIS Fatigue T-score [20] to capture symptom intensity and impact. Pain sensitivity was assessed using a pressure algometer applied over the paraspinal muscles, while pain interference was quantified using a standardized 0–10 numeric scale [21]. The SF-36 Physical Function subscale was used to assess global physical function, with lower scores indicating greater disability [22]. Higher PROMIS fatigue T-scores reflect greater severity of fatigue, and lower SF-36 physical function scores indicate greater disability. These measures served as potential confounders in multivariate analyses and were selected based on established associations with balance, strength, and post-COVID impairments reported in prior studies.

### Data collection instruments and procedures

All assessments were performed in a dedicated biomechanics laboratory under standardized environmental conditions, with temperature and lighting controlled to prevent external sensory influences. Data collection was carried out by a licensed physiotherapist trained in neuromuscular assessment protocols. Before testing, participants underwent a brief familiarization session to reduce anxiety and minimize learning effects. Equipment calibration was conducted daily to ensure data accuracy, and all procedures followed pre-specified standard operating procedures. To reduce measurement bias, the examiner was blinded to participant group allocation, and data were cross verified by a second examiner.

### Sample size calculation

The sample size for this study was determined using G*Power version 3.1.9.7. Based on a moderate effect size (Cohen’s d = 0.5) derived from previously published studies investigating balance and neuromuscular outcomes in post-COVID and related clinical populations, an alpha level of 0.05, and a desired statistical power of 0.80, a total sample size of 128 participants (64 per group) was required to detect significant between-group differences using independent samples t-tests. No pilot data were available for the present study. These parameters were selected to ensure sufficient power for both the primary and secondary analyses proposed in the study.

### Data analysis

Statistical analyses were performed using SPSS version 24.0 (IBM Corp., Armonk, NY, USA). Data normality was assessed using the Shapiro–Wilk test, and all continuous variables were normally distributed (p > 0.05). Homogeneity of variance was evaluated using Levene’s test and was satisfied for all primary outcome measures (p > 0.05). Assumptions of linearity and homoscedasticity were further verified through scatterplot inspection and residual analyses. As all assumptions for parametric testing were met, parametric statistical procedures were applied throughout the study. Between-group differences in postural control parameters, trunk range of motion (ROM), and isometric trunk muscle strength were examined using independent-samples t-tests. Associations between postural control measures and trunk mobility, trunk muscle strength, fatigue, pain sensitivity, and physical function within the Long COVID group were evaluated using Pearson’s correlation coefficients. Statistical significance was set at a two-tailed p-value < 0.05. For multivariate analyses, the stability index was selected as the dependent variable because it represents a comprehensive measure of postural control derived from computerized posturography. Sway area and sway velocity were analyzed as secondary postural outcomes but were not included as additional regression endpoints to avoid redundancy among closely related balance measures. Multiple linear regression analysis was performed to identify independent predictors of postural stability. To control for the potential inflation of Type I error resulting from multiple comparisons, False Discovery Rate (FDR) correction was applied using the Benjamini–Hochberg procedure. Both unadjusted and FDR-adjusted p-values were evaluated where appropriate, and all significant findings remained significant following FDR correction. Multicollinearity among regression predictors was assessed using tolerance and Variance Inflation Factor (VIF) statistics, with tolerance values > 0.20 and VIF values < 5 indicating acceptable levels of collinearity.

## Results

Compared to matched healthy controls, individuals with Long COVID demonstrated significantly greater fatigue, heightened pain sensitivity, and reduced physical function, as indicated by markedly higher Fatigue Severity Scale scores (p < 0.001), lower pain pressure thresholds (p < 0.001), and diminished SF-36 physical function scores (p < 0.001), respectively (Table 1). These clinically meaningful differences were not attributable to demographic or anthropometric factors, as there were no significant group differences in age, sex, height, weight, or body mass index (all p > 0.05). Additionally, while systolic blood pressure was modestly elevated in the Long COVID group (p = 0.045), diastolic pressure and resting heart rate did not differ significantly between groups.

**Table 1:** Demographic and Clinical Characteristics of Participants.

Variable	Long COVID Group (n = 64)	Control Group (n = 64)	p-value
Age (years)	45.67 ± 10.23	44.12 ± 9.87	0.412
Sex (Male/Female)	28/36	29/35	0.847
Height (cm)	167.45 ± 8.76	168.13 ± 9.23	0.568
Weight (kg)	72.34 ± 12.45	70.56 ± 11.87	0.342
Body Mass Index (kg/m²)	25.83 ± 3.54	24.89 ± 3.12	0.109
Time Since COVID Diagnosis (months)	9.34 ± 3.21	—	—
Fatigue Severity Scale (FSS) Score	5.72 ± 1.03	2.34 ± 0.98	<0.001
Pain Pressure Threshold (kg/cm²)	3.21 ± 0.65	4.12 ± 0.57	<0.001
SF-36 Physical Function Score	62.35 ± 10.45	81.76 ± 9.65	<0.001
Systolic Blood Pressure (mmHg)	126.45 ± 12.34	122.67 ± 10.98	0.045
Diastolic Blood Pressure (mmHg)	78.34 ± 9.87	76.23 ± 8.76	0.216
Heart Rate (bpm)	82.45 ± 11.32	78.32 ± 10.45	0.083

FSS: Fatigue Severity Scale; SF-36: Short Form-36; kg: kilogram; cm: centimeter; kg/m^2^: kilograms per square meter; bpm: beats per minute; mmHg: millimeters of mercury.

Adults with Long COVID demonstrated significant impairments in both postural control and trunk biomechanics compared to matched healthy controls, as reflected by increased sway area and sway velocity across all sensory conditions, along with a reduced stability index (Table 2). These balance deficits were accompanied by notable limitations in trunk range of motion, including reduced flexion and extension, as well as significantly lower isometric strength of trunk flexor and extensor muscles. The magnitude and consistency of these differences—each reaching statistical significance (p-values ≤ 0.003)—suggest clinically meaningful disruptions in neuromuscular control and biomechanical function among individuals with post-acute sequelae of COVID-19. All principal between-group differences remained statistically significant following Benjamini–Hochberg False Discovery Rate adjustment, supporting the robustness of the observed findings. Effect size estimates further indicated that these differences were of moderate to large magnitude across most postural control and trunk function measures.

**Table 2:** Postural Control, Trunk Range of Motion, and Strength Measures.

Variable	Long COVID Group (Mean ± SD)	Control Group (Mean ± SD)	Mean Difference	95% CI	t-value	p-value
Sway Area (cm²) – EO, Firm	3.45 ± 1.12	2.87 ± 1.08	0.58	0.20 to 0.96	2.98	0.003
Sway Area (cm²) – EC, Firm	4.76 ± 1.34	3.95 ± 1.21	0.81	0.37 to 1.25	3.59	<0.001
Sway Area (cm²) – EO, Foam	6.89 ± 1.56	5.56 ± 1.43	1.33	0.81 to 1.85	5.03	<0.001
Sway Area (cm²) – EC, Foam	8.12 ± 1.87	6.01 ± 1.65	2.11	1.50 to 2.72	6.77	<0.001
Sway Velocity (cm/s)	1.23 ± 0.34	0.98 ± 0.29	0.25	0.14 to 0.36	4.48	<0.001
Stability Index	78.45 ± 5.67	83.21 ± 6.12	−4.76	−6.80 to −2.72	−4.56	<0.001
Trunk Flexion ROM (°)	52.34 ± 6.78	56.78 ± 5.89	−4.44	−6.64 to −2.24	−3.95	<0.001
Trunk Extension ROM (°)	41.56 ± 5.43	45.67 ± 4.98	−4.11	−5.92 to −2.30	−4.46	<0.001
Trunk Flexor Strength (N)	158.23 ± 21.45	174.34 ± 19.87	−16.11	−23.27 to −8.95	−4.41	<0.001
Trunk Extensor Strength (N)	176.45 ± 23.67	192.67 ± 20.45	−16.22	−23.88 to −8.56	−4.15	<0.001

ROM: Range of Motion; N: Newton; cm^2^: square centimeters; cm/s: centimeters per second; °: degrees; SD: Standard Deviation; CI: Confidence Interval; EO: Eyes Open; EC: Eyes Closed; t-value: test statistic for t-test.

**Fig 2:**
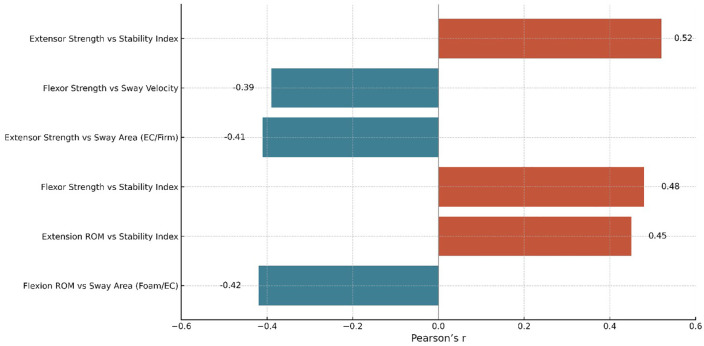
Heatmap of Correlations Between Trunk Measures and Postural Control Parameters in Individuals with Long COVID.

In adults with Long COVID, reduced trunk mobility and diminished isometric strength were significantly correlated with impaired postural control, as evidenced by several moderate associations (Fig 2). Notably, trunk flexion range of motion was negatively correlated with sway area on foam with eyes closed (r = –0.42, p = 0.002), and trunk extension range of motion showed a positive correlation with the stability index (r = 0.45, p = 0.001). Trunk extensor strength demonstrated a strong positive association with the stability index (r = 0.52, p < 0.001) and a negative association with sway area under eyes-closed conditions (r = –0.41, p = 0.003). Additionally, higher trunk flexor strength was associated with both better stability (r = 0.48, p < 0.001) and lower sway velocity (r = –0.39, p = 0.005). These results indicate that compromised trunk function—both mobility and strength—is meaningfully associated with reduced balance control in individuals with Long COVID.

Individuals with Long COVID exhibited significantly higher fatigue levels, greater pain sensitivity, and poorer physical function compared to healthy controls across all measured domains (Table 3). The Fatigue Severity Scale score was markedly elevated in the Long COVID group (5.72 ± 1.03 vs. 2.34 ± 0.98, p < 0.001), accompanied by a higher PROMIS Fatigue T-score (63.42 ± 7.84 vs. 47.23 ± 6.78, p < 0.001), reflecting substantial perceived fatigue. Pain sensitivity was increased, as indicated by a significantly lower pain pressure threshold over the paraspinal region (3.21 ± 0.65 kg/cm² vs. 4.12 ± 0.57 kg/cm², p < 0.001). Pain interference scores were substantially higher (5.23 ± 1.34 vs. 2.01 ± 1.12, p < 0.001), suggesting a greater impact of pain on daily activities. Functional status, as measured by the SF-36 physical function score, was also significantly reduced in the Long COVID group (62.35 ± 10.45 vs. 81.76 ± 9.65, p < 0.001), indicating notable limitations in physical capability.

**Table 3:** Fatigue, Pain Sensitivity, and Functional Scores in Long COVID and Control Groups.

Measure	Long COVID (Mean ± SD)	Control (Mean ± SD)	Mean Difference	95% CI	t-value	p-value
Fatigue Severity Scale (FSS) Score	5.72 ± 1.03	2.34 ± 0.98	3.38	2.99 to 3.77	16.34	<0.001
Pain Pressure Threshold (kg/cm²) – Paraspinal	3.21 ± 0.65	4.12 ± 0.57	−0.91	−1.12 to −0.70	−8.51	<0.001
SF-36 Physical Function Score	62.35 ± 10.45	81.76 ± 9.65	−19.41	−22.95 to −15.87	−10.21	<0.001
PROMIS Fatigue T-score	63.42 ± 7.84	47.23 ± 6.78	16.19	13.76 to 18.62	12.45	<0.001
Pain Interference Score (0–10 scale)	5.23 ± 1.34	2.01 ± 1.12	3.22	2.78 to 3.66	13.87	<0.001

FSS: Fatigue Severity Scale; SD: Standard Deviation; CI: Confidence Interval; SF-36: Short Form Health Survey; PROMIS: Patient-Reported Outcomes Measurement Information System; kg/cm^2^: kilograms per square centimeter.

Note: P-values were additionally evaluated using the Benjamini–Hochberg False Discovery Rate correction for multiple comparisons.

**Fig 3:**
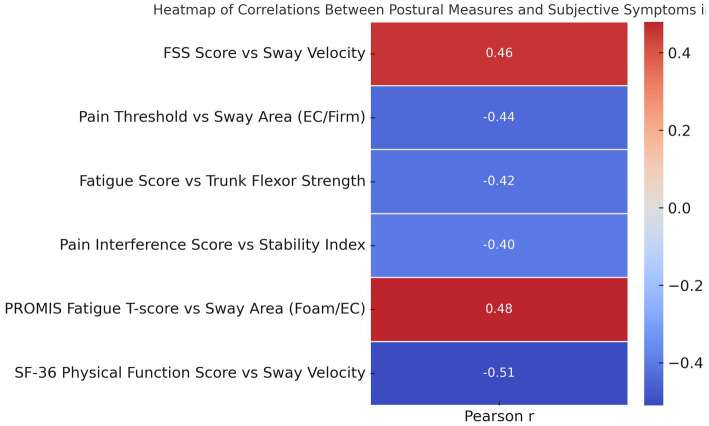
Heatmap of Correlations Between Postural Measures and Subjective Symptoms in Individuals with Long COVID.

In the Long COVID group, greater fatigue, pain sensitivity, and reduced physical function were significantly associated with impaired postural control and reduced trunk strength (Fig 3). Higher Fatigue Severity Scale scores correlated with increased sway velocity (r = 0.46, p = 0.001), and greater PROMIS Fatigue T-scores were positively associated with sway area under challenging sensory conditions (foam surface with eyes closed; r = 0.48, p = 0.001). Lower pain pressure thresholds were related to increased sway area (r = –0.44, p = 0.002), while greater pain interference corresponded to reduced postural control (r = –0.40, p = 0.004). Additionally, higher fatigue was associated with decreased trunk flexor strength (r = –0.42, p = 0.003), and lower SF-36 physical function scores were linked to elevated sway velocity (r = –0.51, p < 0.001), suggesting a consistent pattern whereby subjective symptom burden aligns with objective deficits in balance and core function.

**Fig 4:**
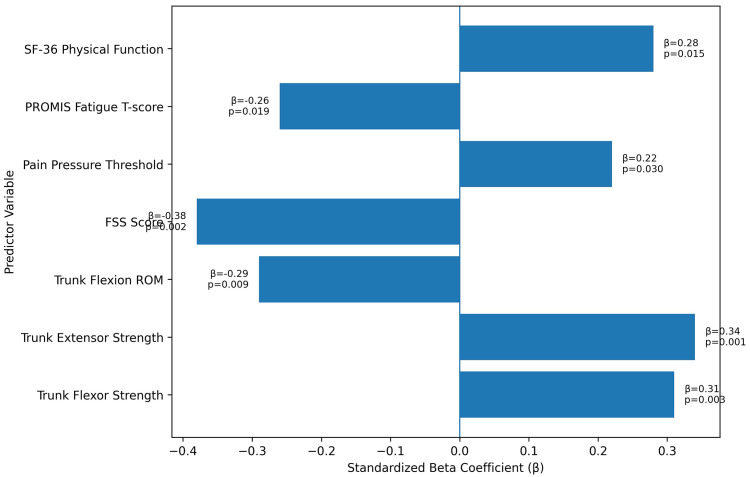
Heatmap of Pairwise Correlations Among Predictor Variables Included in the Correlation and Regression Analyses.

Multivariate linear regression analysis identified several significant predictors of postural instability, as measured by the stability index, in individuals with Long COVID (Table 4). Greater trunk flexor and extensor strength were associated with improved postural control (β = 0.31, p = 0.003 and β = 0.34, p = 0.001, respectively), while reduced trunk flexion range of motion emerged as a significant negative predictor (β = –0.29, p = 0.009). Subjective symptoms also contributed meaningfully to balance impairment: higher fatigue severity (FSS score) (β = –0.38, p = 0.002) and elevated PROMIS Fatigue T-scores (β = –0.26, p = 0.019) were independently associated with poorer stability. Additionally, greater pain pressure thresholds and higher SF-36 physical function scores were positive predictors of postural control (β = 0.28, p = 0.015; β = 0.22, p = 0.030, respectively). These findings highlight the multifactorial nature of balance impairments in Long COVID, integrating both biomechanical and patient-reported dimensions. To further characterize the relationships among predictor variables, pairwise correlation analyses were performed and visualized using a correlation heatmap (Fig 4). Although several variables showed moderate correlations with related aspects of neuromuscular performance and symptom burden, the magnitudes of these correlations did not indicate substantial redundancy among predictors. Effect size analyses demonstrated moderate-to-large effects for most outcomes related to postural control, trunk function, fatigue, pain, and physical function, supporting the clinical relevance of the observed group differences. Assessment of multicollinearity showed acceptable tolerance and VIF values for all predictors in the regression model, indicating that the reported regression coefficients were not substantially influenced by multicollinearity among the independent variables.

**Table 4:** Multivariate Linear Regression Predicting Postural Instability (Stability Index) in Long COVID Group.

Predictor Variable	Unstandardized B	Standard Error	Standardized Beta (β)	95% CI	t-value	p-value
Trunk Flexor Strength (N)	0.028	0.009	0.31	0.010 to 0.046	3.11	0.003
Trunk Extensor Strength (N)	0.032	0.01	0.34	0.013 to 0.051	3.32	0.001
Trunk Flexion ROM (°)	−0.056	0.021	−0.29	−0.098 to −0.014	−2.67	0.009
FSS Score	−0.421	0.134	−0.38	−0.686 to −0.156	−3.14	0.002
Pain Pressure Threshold (kg/cm²)	0.215	0.097	0.22	0.022 to 0.408	2.21	0.03
PROMIS Fatigue T-score	−0.138	0.058	−0.26	−0.253 to −0.023	−2.39	0.019
SF-36 Physical Function Score	0.112	0.045	0.28	0.023 to 0.201	2.48	0.015

ROM: Range of Motion; FSS: Fatigue Severity Scale; PROMIS: Patient-Reported Outcomes Measurement Information System; SF-36: Short Form Health Survey; CI: Confidence Interval; N: Newton; °: Degrees; kg/cm²: kilograms per square centimeter. *The final regression model demonstrated an acceptable fit (Adjusted R² = 0.53, F (7, 56) = 11.42, p < 0.001). VIF values ranged from 1.28 to 2.34, and tolerance values ranged from 0.43 to 0.78, indicating no problematic multicollinearity.*

## Discussion

This study aimed to examine postural control impairments and their connection with trunk biomechanics and subjective symptom severity in adults with Long COVID. Using a combination of computerized posturography, clinical biomechanical assessments, and validated patient-reported outcome measures, the analysis showed significant deficits in static balance performance among individuals with Long COVID compared to matched healthy controls. These balance issues were also linked to reduced trunk range of motion and decreased isometric trunk muscle strength, with notable correlations suggesting that reduced trunk function was significantly associated with postural instability. Moreover, higher levels of fatigue, pain sensitivity, and perceived functional limitations were consistently associated with poorer postural control, highlighting the complex relationship between physical impairments and symptom burden in this group. Multivariate analysis confirmed that both biomechanical and subjective variables independently predicted balance performance, emphasizing the multifaceted nature of postural instability in Long COVID and the importance of comprehensive assessment and targeted treatment strategies. Collectively, these findings suggest that both trunk biomechanics and symptom burden contribute independently to balance impairments in Long COVID, supporting the need for multidimensional rehabilitation approaches. The stability index was selected for multivariate modeling because it reflects overall balance performance and is conceptually broader than individual posturographic measures such as sway area or sway velocity. While these measures capture specific components of postural control, they are inherently related and may share common variance. The absence of problematic multicollinearity among predictors suggests that trunk biomechanics, fatigue, pain-related measures, and physical function contributed distinct information regarding postural stability rather than simply reflecting a single underlying construct. These results suggest that trunk biomechanics and symptom burden contribute independently to postural control deficits rather than reflecting substantial overlap among the measured constructs.

The observed impairments in postural control among individuals with Long COVID, as shown by increased sway area, higher sway velocity, and a lower stability index, suggest significant deficits in sensory integration and neuromuscular regulation [5]. These findings were most evident under eyes-closed and foam surface conditions, indicating an altered reliance on proprioceptive and vestibular inputs [5]. From a clinical perspective, these findings suggest that individuals with Long COVID may have greater difficulty maintaining postural stability in environments with limited visual information or unstable surfaces, potentially increasing the risk of balance loss and falls. However, these results should not be interpreted as an indication to avoid such environments altogether. Rather, they support the incorporation of progressively graded balance training that safely challenges visual, proprioceptive, and vestibular systems to enhance sensory integration, improve adaptability to real-world conditions, and reduce fall risk. Clinicians may also consider providing education on environmental hazards and implementing appropriate safety measures during activities in visually impaired or unstable settings. The moderate-to-strong correlations between trunk flexor and extensor strength and postural parameters, along with reduced trunk range of motion, support the essential role of trunk biomechanics in maintaining postural stability [23,24]. Trunk extensor strength, in particular, showed the strongest positive association with the stability index, whereas decreased trunk flexion range of motion was associated with increased postural sway [25,26]. Regression analysis identified trunk strength, mobility, and fatigue as variables independently associated with postural instability, emphasizing their clinical relevance. The final regression model explained a substantial proportion of the variance in postural stability (adjusted R^2^ = 0.53), indicating that trunk biomechanical characteristics and symptom-related factors collectively accounted for a meaningful share of the variability in balance performance in individuals with Long COVID. This finding further supports the multifactorial nature of postural dysfunction in this population.

These results are aligned with existing literature reporting balance disturbances in post-viral fatigue and COVID-19 syndromes. De Sousa et al. [26] reported altered postural sway and increased instability in post-acute COVID populations, particularly under sensory-challenging conditions. In contrast, Baratta et al. [27] highlighted that impaired trunk control compromises balance in fatigue-related disorders. Similarly, Wrisley et al.[28] emphasized the influence of trunk muscle weakness and mobility limitations on sensorimotor integration and functional stability. Miana et al. [29] further observed that reduced trunk range and neuromuscular activation contribute to impaired core stabilization in post-viral fatigue syndromes, and Laming-van Eijk et al. [30] documented diminished trunk strength in post-COVID patients, attributing this to inactivity and myopathic changes. In addition, Eskandari et al. [31] demonstrated that critical illness survivors experience long-lasting trunk dysfunction directly linked to delayed postural recovery and elevated fall risk. More recently, Pazdro-Zastawny et al. [32] found balance deficits in Long COVID patients comparable to those in older adults with vestibular dysfunction, reinforcing the role of multisystem disruption. Collectively, these converging findings support the present results and highlight the clinical relevance of systematically evaluating trunk biomechanics and integrating trunk-focused rehabilitation strategies in the management of postural instability in Long COVID.

The association between subjective symptoms and postural control in individuals with Long COVID was evident in consistent correlations among fatigue, pain sensitivity, and perceived functional limitations and key balance parameters. Higher fatigue scores, on both the Fatigue Severity Scale and the PROMIS Fatigue T-score, were significantly associated with increased sway velocity and sway area, whereas greater pain interference and lower pain pressure thresholds were associated with poorer stability and greater postural sway [33]. Lower Physical function, as measured by the SF-36, was also inversely correlated with balance performance. These relationships remained significant in the multivariate regression analysis, where fatigue, pain sensitivity, and functional status remained independently associated with postural instability. These findings are in agreement with previous research by Wrisley et al. [28], who demonstrated that subjective fatigue in Long COVID patients was associated with impaired sensorimotor integration and delayed postural responses. Similarly, Khoja et al. [34] reported that chronic musculoskeletal pain and reduced physical function significantly contributed to diminished balance capacity in post-COVID populations. Moreover, findings by Hayes et al. [35] underscored that fatigue and physical deconditioning were primary contributors to reduced functional mobility and postural control in long-term COVID-19 cases. Together, these results highlight the relevance of integrating subjective symptom profiles into clinical assessments of balance in Long COVID populations.

### Clinical significance

The clinical significance of this study lies in its comprehensive identification of modifiable neuromuscular and symptom-related factors associated with postural instability in individuals with Long COVID. By demonstrating that impairments in trunk mobility and strength are not only prevalent but also significantly associated with objective balance deficits, the findings underscore the importance of incorporating targeted trunk-focused rehabilitation strategies to restore postural control. Moreover, the observed relationships among fatigue, pain sensitivity, functional limitations, and balance performance highlight the need to integrate symptom management—particularly fatigue and musculoskeletal pain—into individualized therapeutic plans. These insights provide a clear foundation for multidisciplinary interventions that address both biomechanical and symptom-driven dimensions of postural dysfunction, ultimately aiming to reduce fall risk, enhance mobility, and improve quality of life in the Long COVID population. The identification of independent associations across biomechanical and symptom-related domains further suggests that rehabilitation programs should not rely on a single therapeutic focus. Instead, integrated interventions targeting trunk mobility, trunk strength, fatigue management, pain-related impairments, and functional capacity may be more effective in addressing the multifactorial nature of postural dysfunction in this population.

### Limitations

Several limitations should be acknowledged when interpreting the findings of this study. First, the cross-sectional design precludes causal inference regarding the relationships among trunk biomechanics, symptom burden, and postural control in individuals with Long COVID. Consequently, the observed associations should not be interpreted as evidence of causality, and longitudinal studies are needed to clarify the temporal and causal relationships among these variables. Second, although clinically relevant and widely validated, self-reported measures of fatigue, pain, and physical function may be influenced by individual perceptions and psychological factors that were not assessed in the present study. Third, the sample consisted exclusively of ambulatory individuals with moderate symptom severity, limiting the generalizability of the findings to individuals with more severe Long COVID, including those with substantial mobility limitations, recent hospitalization, or complex multisystem involvement. Additionally, although robust clinical and biomechanical assessment tools were employed, no direct neurophysiological or vestibular evaluations were performed. Therefore, the proposed mechanisms related to altered sensory integration, neuromuscular regulation, and postural dysfunction remain speculative, and the specific neural, vestibular, or sensorimotor pathways underlying the observed impairments cannot be determined from the present data. Future studies incorporating electromyography, vestibular function testing, and other neurophysiological assessments are warranted to further elucidate these mechanisms. Furthermore, while several clinically relevant covariates were included in the regression analyses, other potentially important factors, such as habitual physical activity, medication use, physical deconditioning, and rehabilitation history, were not systematically assessed. The omission of these variables may have contributed to residual confounding and should be considered when interpreting the reported associations. Future research should employ longitudinal designs to examine recovery trajectories, evaluate the effectiveness of trunk-focused rehabilitation interventions, and investigate the neurophysiological mechanisms underlying postural control deficits in Long COVID. The inclusion of more diverse patient populations, objective physical activity monitoring, and advanced analytical approaches may further enhance understanding of the multifactorial nature of balance impairments and improve the translational relevance of future studies.

## Conclusion

This study demonstrates that adults with Long COVID exhibit significant impairments in postural control, closely associated with reduced trunk mobility, decreased isometric trunk strength, and elevated fatigue, pain sensitivity, and functional limitations. Objective measures of balance, including sway area, sway velocity, and stability index, were consistently correlated with both biomechanical deficits and symptom burden. Multivariate analysis demonstrated that trunk function and subjective symptoms were independently associated with postural instability. These findings highlight the multifactorial nature of balance impairments in Long COVID and support the clinical relevance of comprehensive assessments that integrate biomechanical evaluation and symptom profiling to inform targeted rehabilitation strategies.
